# Feasibility of Sentinel Lymph Node Biopsy in Breast Cancer Patients with Axillary Conversion after Neoadjuvant Chemotherapy—A Single-Tertiary Centre Experience and Review of the Literature

**DOI:** 10.3390/diagnostics13183000

**Published:** 2023-09-20

**Authors:** Alexandra Maria Lazar, Mario-Demian Mutuleanu, Paula Monica Spiridon, Cristian Ioan Bordea, Tatiana Lucia Suta, Alexandru Blidaru, Mirela Gherghe

**Affiliations:** 1 Carcinogenesis and Molecular Biology Department, Institute of Oncology “Prof. Dr. Alexandru Trestioreanu”, 022328 Bucharest, Romania; 2 Nuclear Medicine Department, University of Medicine and Pharmacy “Carol Davila”, 050474 Bucharest, Romania; 3 Nuclear Medicine Department, Institute of Oncology “Prof. Dr. Alexandru Trestioreanu”, 022328 Bucharest, Romania; 4 Surgical Oncology Department, University of Medicine and Pharmacy “Carol Davila”, 050474 Bucharest, Romania; 5 Surgical Oncology Department, Institute of Oncology “Prof. Dr. Alexandru Trestioreanu”, 022328 Bucharest, Romania

**Keywords:** lymphoscintigraphy, sentinel lymph node biopsy, breast cancer, neoadjuvant chemotherapy

## Abstract

(1) Introduction: Sentinel lymph node biopsy (SLNB) is widely used in breast cancer patients who undergo neoadjuvant chemotherapy (NAC), replacing axillary lymph node dissection. While commonly accepted for cN0 patients, its role in cN1/2 patients remains controversial. Our study aims to investigate the role of SLNB in BC patients who underwent prior NAC and compare our results to those of other studies presented in the literature. (2) Materials and methods: Our retrospective study included 102 breast cancer patients who received NAC before ^99m^Tc-albumin Nanocolloid SLN mapping and SLNB was performed, completed or not with axillary dissection. A review based on the PRISMA statement was also carried out, encompassing 20 studies. (3) Results: The lymphoscintigraphy performed after the administration of NAC presented an identification rate (IR) of 93.13%. IR for SLNB was 94.11%, with a false-negative rate (FNR) of 7.4%. After a median follow-up of 31.3 months, we obtained a distant disease-free survival rate of 98%. The results obtained by other groups were similar to those of our study, presenting IR in the range 80.8–96.8%, with FNR varying from 0 to 22%. (4) Conclusions: on conclusion, SLNB can accurately determine the lymph node status, with an acceptable FNR and maintain its expected prognostic role with low recurrence rates, and our results are comparable to those obtained by other studies.

## 1. Introduction

Breast cancer (BC) is the most frequently diagnosed cancer and the leading cause of cancer-related death among women, with an estimated incidence of 11.7% and more than 500,000 deaths attributed to it worldwide [1]. The widespread and rapid development of diagnostic methods and primary systemic therapy have revolutionised the management of patients diagnosed with BC. Under the circumstances of the great therapeutic response of these new treatments, the next major concern and future direction for BC patient management is to reduce the extent of the needed surgical procedures while improving the patients’ quality of life [2,3]. Some recent studies even proposed chimeric antigen receptor therapy for the treatment of BC [4]. The advancements in pre-operative chemotherapy for the initial management of advanced-stage breast tumours presented the question of whether sentinel lymph node biopsy (SLNB) could represent a safer option, with fewer side effects in this subset of patients.

Neoadjuvant chemotherapy (NAC) has been implemented to convert originally inoperable tumours into operable tumours while also offering the possibility to appraise the degree of treatment response [5]. Moreover, NAC is frequently administered to patients with operable but large BC in an attempt to shrink the tumour size, lower its clinical stage and increase the rate of breast-conserving surgery [2,6,7].

The histopathological status of the axillar and locoregional lymph nodes is an important prognostic factor in breast cancer patients and plays a significant role in guiding future treatment decisions [8,9]. NAC has been proven to successfully downstage up to 40% of pre-chemotherapy-documented axillary lymph node (ALN) metastases and even eradicate biopsy-proven ALN metastases in 32% of BC patients [10]. In these patients, the increasingly pivotal role played by NAC has had an important impact on the extent of axillary surgical procedures, paving the way to a more “targeted” procedure in women with node-positive BC who convert to negative lymph nodes after NAC [11]. The SLNB technique is a minimally invasive approach, which provides the same staging information that can be gathered through ALN dissection (ALND), but with minimal associated morbidities, usually resulting in a better quality of life [12,13]. This approach offers several advantages, such as reducing the psychological impact associated with radical treatments and minimizing aesthetic and functional impairments.

Identifying the SLN for excision during SLNB is usually carried out through the radioisotopic technique. Radioisotopic mapping is a nuclear medicine technique based on the principle of lymphatic dissemination of the cancerous cells from the primary tumour site to the lymph node stations via the lymphatic pathways [13]. Sentinel lymph nodes (SLNs) are the regional nodes that directly drain lymph from the primary tumour, being the first nodes to receive lymph-borne metastatic cells [14]. Although no imaging modality is accurate enough to detect lymph node metastases in early-stage BC (stages I–II), SLN biopsy is a highly reliable method of screening axillary nodes and identifying metastatic and micrometastatic disease in regional lymphatic nodes, nowadays being considered “the gold standard” for axillary staging in early BC patients with clinically negative lymph nodes [15,16]. Histopathological and immunohistochemical tests are usually performed on the excised SLN to determine the degree of infiltration with tumoral cells in order to establish the further therapeutic procedures required [17,18,19].

There are some clinical circumstances where SLNB is recommended: if the primary tumour is T1, T2 or ductal carcinoma in situ; when ductal carcinoma in situ management includes mastectomy; if the patient is male; in case of obesity or older age; or when pre-operative systemic therapy is planned [16]. Nevertheless, the role of SLNB after NAC remains a subject of debate. Even if the NCCN (National Comprehensive Cancer Network) guidelines recommend this procedure in patients who receive NAC, the correct indication for SLNB after NAC in BC is still controversial, particularly in patients with clinically involved axilla that shift to being clinically negative after treatment [20,21].

The efficacy of SLNB following NAC has not yet been firmly established due to the impact of NAC on intramammary lymphatic vessels and axillary tissues. These effects, including fibrosis, fat necrosis and the formation of granulation tissue, have the potential to modify lymphatic drainage patterns, thereby affecting the identification rate (IR) and accuracy of subsequent lymphatic mapping [22]. Moreover, histological changes in the breast and draining lymphatics caused by chemotherapeutic agents can contribute to a low success rate and a high false-negative rate in SLNB following neoadjuvant chemotherapy [22,23,24].

The aim of our study is to present the experience gathered by our tertiary centre via radiosotopic mapping, followed by SLNB, in BC patients who received NAC prior to the procedure, as well as giving an overview of this technique in other institutions that followed the same conditions.

## 2. Materials and Methods

### 2.1. Study

#### 2.1.1. Patient Selection

We performed a retrospective study in which data were collected from 131 patients diagnosed with breast cancer, who received neoadjuvant chemotherapy before undergoing a lymphoscintigraphy treatment with [^99m^Tc]-albumin nanocolloid, between January 2016 and May 2023 in the Department of Nuclear Medicine of the Institute of Oncology “Prof. Dr. Alexandru Trestioreanu”, Bucharest, Romania.

The patient inclusion criteria were as follows: (1) histopathologically confirmed BC; (2) the implementation of NAC before SLN mapping; (3) SLN mapping performed with ^99m^Tc-albumin nanocolloid; (4) TNM staging before and after NAC.

Information on the histopathological type included the following immunohistochemical tumoural markers: estrogen receptor (ER), progesterone receptor (PR), human epidermal growth factor receptor 2 (HER 2), proliferation index ki67 and tumour/node/metastasis (TNM) staging before and after chemotherapy, and the chemotherapy protocols used were collected from the medical records of the patients.

Considering the selection criteria, 102 patients fulfilled the requirements and were enrolled in our study.

#### 2.1.2. Nanocolloidal Albumin (Nanocoll^®^) Preparation

The preparation of ^99m^Tc-albumin nanocolloid was performed according to the labelling instruction provided by the manufacturer (ROTOP Pharmaka GmbH, Dresden, Germany), which will be further described.

The formation of the ^99m^Tc-nano-sized albumin colloid depends on a sufficient content of tin in the reduced state. Oxidation can affect the quality of the preparation; this issue is why an air inlet has to be strictly avoided. The first step was to place the vial in a suitable lead shield and add 1–5 mL of sodium pertechnetate solution (185 MBq to 5.5 GBq) into the vial using a sterile syringe. The next step was to dissolve the dry substance via repeated inverting, before allowing it to stand for 10 min at room temperature.

^99m^Tc-labelled human serum albumin colloid can be stored at room temperature and used within 6 h [25].

#### 2.1.3. Imaging Acquisition Technique

No special preparation was necessary prior to the investigation.

For detection, SLN mapping was performed 18–20 h before surgery. Each patient was administered a volume of 0.3–0.5 mL containing a dose of 18–37 MBq of ^99m^Tc-albumin nanocolloid via peritumoural injection under ultrasound guidance. In patients with complete tumoural remission after NAC, the colloid was injected around the intratumourally inserted clip at the time of the core biopsy.

The gamma camera used for exams was a dual-head Discovery 670 DR single-photon emission-computed tomography-computed tomography (SPECT/CT) system (General Electric Healthcare, Chicago, Illinois, United States of America) with low-energy high-resolution (LEHR) collimators. The examination protocol included an acquisition of static images in different incidents, most commonly the anterior, lateral and left/right–oblique–anterior at 45°. The planar scan was acquired 30 min after the injection of the radiopharmaceutical and, as needed, at up to 2 h using the following parameters: the detectors were placed in H position, with detector 1 covered with a flood phantom with an activity of 1–1.5 mCi ^99m^Tc-pertechnetate, the matrix size being 128 × 128, each image capturing 500,000 counts and the energy window centered on the 140-kiloelectronvolt photopeak of ^99m^Tc.

In cases where multiple or overlapping SLNs were visualised on the planar scan, given the difficulties in correctly distinguishing and localising them, a SPECT/CT scan was conducted to ensure the accurate mapping of these nodes. SPECT/CT imaging was executed using a standard protocol: a 128 × 128 matrix size, a step-and-shoot rotation time of 30 s/view (for a total of 120 views) and a dual energy window (140 keV ± 10% and 120 keV ± 5%). After the SPECT acquisition, a low-dose CT scan was performed, maintaining the patient in the same position, at 120 keV and 40–340 mA. CT images were acquired using a standard 3.75 slice thickness and reconstructed with reconstruction filter provided by the vendor.

The acquired SPECT/CT data were analysed on the Q.Volumetrix software (Xeleris 4.0 workstation) provided by the manufacturer, using the ordered subset expectation maximisation (OSEM) iterative reconstruction algorithm (8 subsets and 10 iterations), with attenuation correction, scatter correction and resolution recovery being performed.

After the scans, a mark on the skin surface was applied at the projection site of the hottest nodes to roughly indicate their position for surgery.

#### 2.1.4. Intraoperative SLN Identification

The identification of the SLN was performed during the surgery using a portable gamma probe, which detects the radioactive tracer present in the SLN, also referred to as the “hot node”. The nodes with the highest uptake were excised and sent for histopathological examination. Following that step, it underwent analysis using paraffin-embedded sections and immunohistochemistry techniques for a precise diagnosis [26].

#### 2.1.5. Statistical Analysis

Data analysis was performed utilizing IBM SPSS Statistics Version 26 (IBM, SPSS, Inc., Chicago, IL, USA, 2019). The data were presented as mean and standard deviation. Statistical tests were performed to ascertain the success rate of lymph node identification following lymphoscintigraphy, along with the subsequent identification and excision of these nodes during the surgical procedure. To compare the results, a paired-samples T-test was employed. Additionally, correlations between lymph node status before and after NAC were established using Pearson’s correlation test. Statistical significance was considered for *p* values < 0.05.

### 2.2. Literature Review

#### 2.2.1. Aims of Review

Our literature review aims to give an overview of the studies investigating the role of SLNB, using radioisotopic mapping for SLN detection in patients who underwent NAC for BC before the investigation.

#### 2.2.2. Search Algorithm

**Figure 1:**
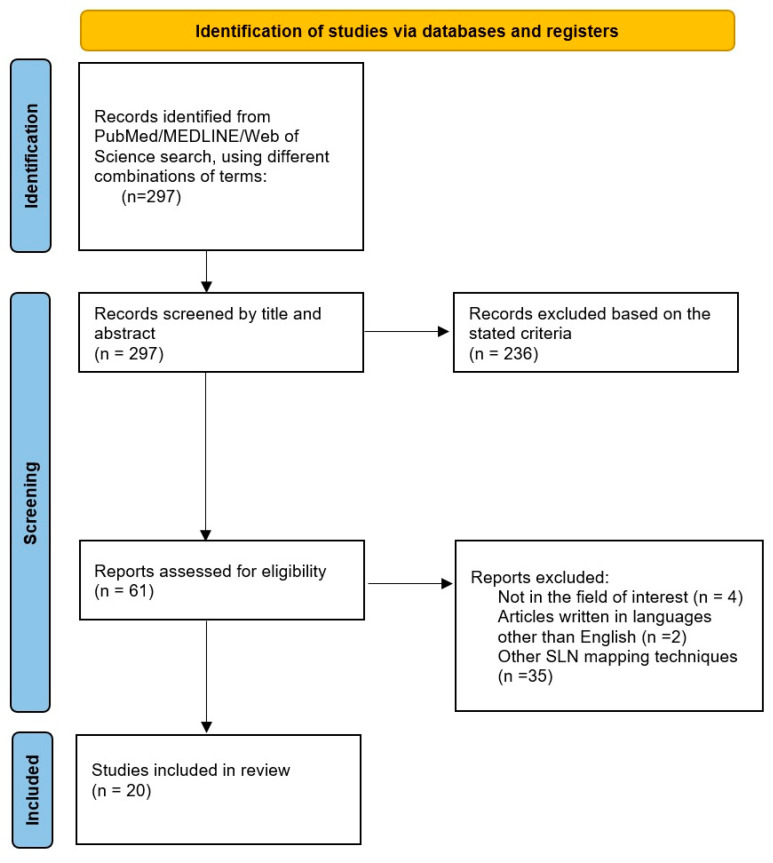
Schematic representation of the process of literature selection for the qualitative review according to the PRISMA statement.

A comprehensive search algorithm in the PubMed, MEDLINE and Web of Science databases was constructed based on the combination of the following terms: “breast cancer”, “neoadjuvant chemotherapy”, “sentinel lymph node biopsy” and “radioisotopic mapping” (Figure 1). No beginning date was applied, and the search was extended up to May 2023. To expand our research, we manually evaluated references from the retrieved articles to search for supplementary useful studies. We followed the Preferred Reporting Items for Systematic Reviews and Meta-Analysis (PRISMA) guidelines to select the relevant studies [27].

The inclusion criteria required the following aspects: (a) articles written in English and (b) investigating patients with breast cancer who performed NAC (c) before the mapping of the SLN with radiolabelled agents. Both prospective and retrospective studies were considered eligible.

The exclusion criteria were as follows: (a) articles not within the field of interest; (b) articles written in languages other than English; (c) case reports or small case series; (d) the use of blue dye (BD), indocyanine green or other substances for SLN mapping (e) in vitro or animal studies; (f) reviews and meta-analysis articles, letters, comments or conference proceedings.

#### 2.2.3. Data Extraction and Synthesis

Among the 297 articles identified after the first search, 61 studies were assessed for eligibility based on the stated inclusion criteria, and 20 studies were finally selected for this qualitative synthesis. Data extracted from each publication included the authors, research design, study reasons, imaging techniques, number of enrolled patients, status of lymph nodes before and after treatment, results of SLN mapping, the rate of SLN identification and study results. A descriptive overview was performed to provide key summary statistics of the information obtained from the papers.

## 3. Results

### 3.1. Study

Between January 2016 and May 2023, 131 patients diagnosed with BC, who received NAC before SLN detection, were treated in our tertiary centre. Being a retrospective study, only 102 of the patients satisfied all of the necessary conditions and were included in our study. The median age of the patients was 55 ± 11.9 years (range 24–77), with a median of 56 years old (range 31–75) in cN0 patients and 54 years old (range 24–77) in cN1/2 patients. All patient characteristics prior to NAC, according to axillary clinical status before the systemic treatment, the stage of the disease and the immunohistochemical characteristics, are summarised in Table 1, Table 2 and Table 3.

At the time of diagnosis, 47 patients had no lymph node involvement (cN0), while 55 individuals were considered as lymph node positive (cN1/2). Different types of NAC, such as anthracyclines, taxanes, anti-HER2 therapies and hormonal therapy in different combinations, were administered according to specific therapeutic protocols. The mean number of chemotherapy cycles in our patient cohort was 7.43, with an average of 7.12 for cN0 patients and 7.70 for those classified as cN1/2 (*p* < 0.05). All patients had their axillary status evaluated through ultrasound after the completion of their NAC regimens.

After NAC completion, 40 patients who were initially classified as cN0 remained ycN0, 42 cN1/2 patients were downstaged to ycN0 status, 10 patients were cN1/2 both before and after treatment and 10 patients presented disease progression (Table 4). Table 5 presents the data regarding the pathological response. In our patients, an axillary pathologic complete response (pCR) rate of 77.36% was discovered.

The best response to NAC was found in patients initially classified as stage II and III, with downstaging observed in 65.15% and 84.61% of cases, respectively. Regarding the patients initially diagnosed as stage I, the percentage of those with downstaging and disease progression was the same, i.e., 22.22%, for each category. However, the majority of patients presented stable disease (55.55%).

In 95 patients, the visualisation of at least one SLN within the initial timeframe of 2 h post-radiotracer administration for lymphoscintigraphy was successful. Among those individuals, 16 patients presented a positive histopathological result after intraoperative identification and excision.

During the lymphoscintigraphy performed after NAC, an average of 1.56 SLNs were identified (with a range of 0–4) within an average time interval of 49.7 min. For patients who had cN0 status at the time of diagnosis, the average number of identified lymph nodes was 1.59 within an average time of 48.9 min. In contrast, for those who had cN1/2 status at the time of diagnosis, the average number of identified SLNs was 1.54 within an average time of 53.6 min. By analysing the data derived from the lymphoscintigraphy results, we found that the rate of SLN identification via lymphoscintigraphy was 93.13%.

**Figure 2:**
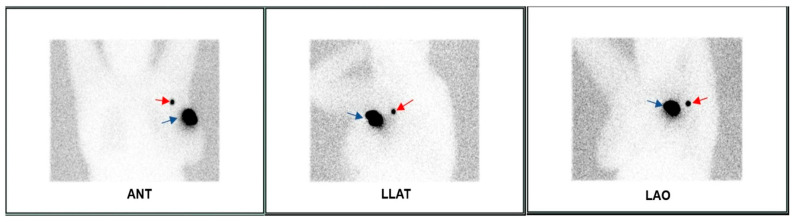
Planar lymphoscintigraphy of 48-year-old patient diagnosed with Stage III invasive ductal carcinoma of breast, showing one left axillary sentinel lymph node (red arrow). Blue arrow represents injection site.

**Figure 3:**
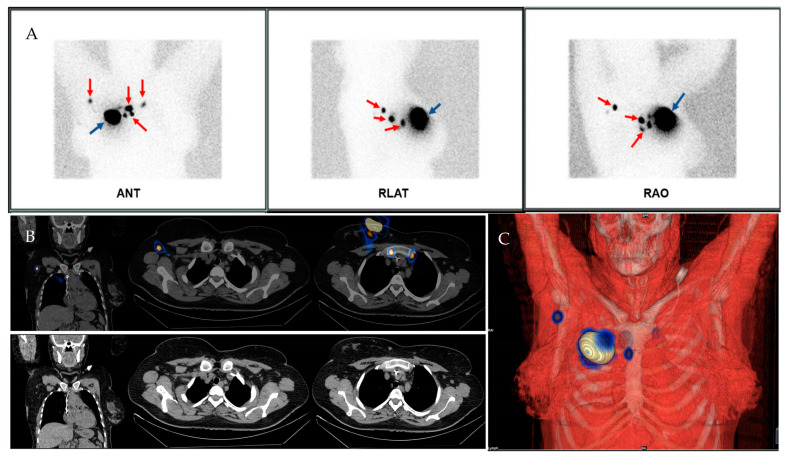
Female patient aged 45 years old diagnosed with Stage II invasive ductal carcinoma of the breast. (**A**) Planar lymphoscintigraphy showed multiple SLNs (red arrows) localised in different lymph node levels and overlapping the injection site (blue arrow). (**B**) For more precise localisation of the SLNs, the patients underwent SPECT/CT examination that revealed lymphatic drainage originating from the peritumoural region into the right axillary region (involving 1 SLN) and the bilateral internal mammary regions (involving two nodes on the right side and one on the left side). Figure (**C**) shows a 3D rendering of the SLNs positions.

Considering the 102 patients included in this study, SLNs were detected using the gamma probe during the surgery and subsequently excised in 96 patients, resulting in a successful IR of 94.11%. A mean of 3.52 SLNs identified during surgery were removed. The average number of lymph nodes found in the group of cN0 patients was 2.97, while for cN1/2 patients, it was 3.92. Five patients required the completion of the planar lymphoscintigraphy scan with a SPECT/CT examination (Figure 2 and Figure 3) to accurately identify the number and location of SLNs, as the distribution of the SLN on the planar image was unclear.

In the case of the seven patients with negative lymphoscintigraphy results, SLNB successfully identified the presence of SLNs, with three of them showing tumour invasion.

After analysing the histopathological examinations of SLNs, it was observed that no presence of metastases was found in 81.37% of patients. Moreov34, 18.62% (n = 19) patients had evidence of tumour invasion at the lymph node levels, with the majority of these patients having been diagnosed with stage II disease and classified as having stable disease following NAC. Regarding the SLN positive patients, seven individuals had initially been diagnosed with cN0 status (Table 6).

In 27 patients, ALND was also performed after SLNB at the surgeon’s consideration following the intraoperative assessment of the axilla. By analysing the histopathological results of these patients, we discovered that in two cases, the SLNB was negative, but tumoural infiltration was present in the other excised lymph nodes, thus resulting in a false-negative rate (FNR) of 7.4% (2/27).

After a median follow-up of 31.3 months (range 5–87 months), recurrence was observed in four patients, two of whom developed hepatic metastases at an average of 66 months after surgery, resulting in a distant disease-free survival of 98%. These two patients had lymph node involvement both before and after NAC. Another patient was diagnosed with residual breast carcinoma 20 months after surgery, despite having N0 status both before and after NAC, while another patient presented with lymph node metastasis 48 months after surgery, resulting in an axillary recurrence rate of 2.1%. In the case of axillary recurrence, regional disease-free survival was also 98%.

### 3.2. Literature Review

Numerous scientific papers have focused on the utility of SLNB in BC patients who received prior NAC for disease downstaging. In our review, we only included the studies using radioisotopes as SLN mapping technique, in agreement with the protocol used in our research. The main characteristics of the studies focused on regarding this matter are summarised in Table 7.

One of the first studies published on the use of SLNB after radioisotope SLN mapping in BC patients priorly treated with NAC was published by Tafra et al. [28] in 2001. Their group investigated the effect of NAC in 29 patients undergoing SLNB for breast cancer compared to those who did not receive NAC. Their results stated an SLN IR of 93% and FNR of 0% in the NAC group, while in the group without prior NAC, the values for the two rates were of 88% and 13%, respectively, showing that prior NAC did not adversely impact the SLNB’s performance.

Shimazu et al. [29], in 2004, enrolled 47 patients diagnosed with BC, who received different protocols of NAC prior to SLNB. They used ^99m^Tc-tin colloid and BD for SLN mapping. Their results showed a successful IR for SLNB of 94% and an FNR of 12.1%, which tended to be higher, although not to a statistically significant extent, among patients with clinically positive axillary lymph nodes before and/or after NAC.

Later, Kinoshita et al. [30] evaluated the feasibility and accuracy of SLNB following NAC in patients with operable BC, enrolling a total of 104 patients with stage II and III BC. SLN could be identified in 97 of 104 patients, resulting in an IR of 93.3%, with an FNR of 10.0%. They also concluded that SLN IR tended to be lower among patients with T4 primary tumours prior to NAC (62.5%).

In 2007, Newman et al. [31] tried to establish the optimal strategy for incorporating lymphatic mapping and SLNB into the management of BC patients receiving NAC. They evaluated 54 consecutive BC patients with biopsy-proven axillary nodal metastases at the time of diagnosis who underwent lymphatic mapping with nodal biopsy, as well as concomitant ALND, after receiving NAC. The authors obtained an IR after delivery of neoadjuvant chemotherapy of 98%, with an FNR of 8.6%.

Ozmen et al. [32], in 2009, aimed to determine the feasibility and accuracy of SLNB after NAC in patients with locally advanced BC, who initially had confirmed positive ALN that became clinically negative after NAC. They investigated 77 patients with locally advanced BC, who underwent SLNB via both BD and radioisotope injection. Their results showed a successful IR for SLNB of 92%, accurately predicting axillary status in 90% of cases, with a false-negative rate of 13.7%. They also found that positive non-SLN involvement was more common in patients with multifocal/multicentric tumours and positive lymphovascular invasion, especially in patients with a larger pathologic tumour size (>2 cm) and positive extra SLN extension.

One of the largest studies investigating patients with BC who underwent NAC radioisotope SLN mapping and SLNB was performed by Pecha et al. [33] in 2011. The study included 343 patients with BC who underwent lymphatic mapping to identify the SLNs. The researchers discovered that at least one SLN was successfully identified and excised in 277 patients, resulting in an overall success rate of 80.8%, influenced by the clinical lymph node status. The FNR obtained by their group was, however, relatively high, i.e., 19.5%, showing that this result occurred when neither lymphatic nor vascular invasion was detected in the tumour. The authors stated a sensitivity of 80.5% and an accuracy of 91.5% for the method.

Another study that researched the feasibility and accuracy of SLNB after the delivery of NAC was conducted by Canavese et al. [34]. The authors evaluated 64 consecutive BC patients with infiltrating BC and clinically positive ALN, who received NAC and subsequent lymphatic mapping through lymphoscintigraphy, SLNB and complete ALND. By evaluating the 106 excised SLNs, they obtained an FNR of 5.1% for SLNB, an SIR of 93.8% and a negative predictive value of 91.3%. The method presented a sensibility of 88.1%, a specificity of 100% and an overall accuracy of 96.7%, making SLNB a suitable technique for assessing the axilla in BC patients after NAC.

Park et al. [35], in a study published in 2013, investigated the diagnostic performance of SLNB after NAC in patients with locally advanced BC with cytology-proven node metastasis at diagnosis. The authors performed SLN mapping through radioisotope technique alone using ^99m^Tc-phytate, identifying a total of 352 SLNs in 169 patients. The successful IR of their technique was 94.9%. They also obtained a sensitivity, an FNR, a negative predictive value and an accuracy for this technique of 78%, 22%, 75.8% and 87%, respectively. The authors stated that SLNB performance was the worst among patients with a single retrieved SLN and best when more than three SLNs were evaluated.

In the same year, another study that studied node-positive breast cancer patients with negative axillary conversion after NAC was conducted by Lee et al. [36]. Their primary goal was to evaluate the feasibility of SLNB in node-positive BC patients with negative axillary conversion after NAC in a prospective clinical trial. The patients were screened using ^18^F-fluorodeoxyglucose positron emission tomography/computed tomography (^18^F-FDG PET/CT), and ultrasonography, and lymphatic mapping was carried out using ^99m^Tc tin colloid. Their results showed a good IR for SLNB (84.3%), with an FNR, a negative predictive value and an accuracy rate of 18.4%, 78% and 87.9%, respectively.

The best study to analyze a specific algorithm for the timing of a standardised SLNB procedure in patients who underwent NAC was the SENTINA study, a four-arm, prospective and multicentre cohort study conducted under the supervision of Kuehn et al. [19]. They included 1737 patients grouped into four arms, depending on the timepoints of the NAC administration and SLNB procedure. ARM C was composed of 592 patients with clinically positive lymph nodes, which underwent NAC before SLNB. They discovered an overall SLNB IR of 80.1%, with a higher IR in patients who received dual-tracer (RI + BD) SLN mapping (87.8%) than in those with RI mapping alone (77.4%). Of 474 patients in arm C who were converted from clinically positive to negative axillary status after neo adjuvant chemotherapy and had a successful sentinel-lymph-node procedure, 248 (52.3%) had ypN0 status and 226 (47.7%) were ypN1. The overall FNR in this arm was 14.2%.

Yagata et al. [37] aimed to evaluate the accuracy of SLNB in detecting ALN metastases in a prospective study. They investigated 95 patients with BC who had confirmed positive lymph nodes prior to NAC and exhibited partial or complete responses to it. Their results revealed an IR of 85.3% for SLN, along with a false-negative rate of 15.7%. Additionally, the study found that the FNR was significantly lower in the HER2-negative group compared to the HER2-positive group.

The study of Kim et al. [38] aimed to assess the feasibility and precision of SLNB while determining whether choosing SLNB alone or SLNB combined with ALND affects axillary recurrence or survival in node-positive BC patients. Data derived from 199 BC patients with positive lymph node, who underwent NAC before lymphoscintigraphy, were used. SLN mapping was performed using both BD and radioisotope injection, resulting a high IR for SLNB of 95.8%, with an FNR of 10%.

Another study that included a large population was carried out by Boughey et al. [23] in their ACOSOG Z1071 (Alliance) trial. They evaluated SLNB effectiveness according to the SLN mapping technique in 756 patients who received NAC for BC prior to the surgical procedure, stating that the use of radiolabelled colloid in combination with BD offers the best IR, i.e., 92.7%, compared to BD or radioisotopes alone.

Andreis et al. [9] aimed to compare the effectiveness of pre-treatment lymphoscintigraphy and post-NAC scan in order to reduce false-negative rates for SLNB in BC patients. The study included 170 consecutive T2-4 cN0-1 M0 BC patients, who underwent sentinel lymphatic mapping at the time of incisional biopsy and then repeated the lymphoscintigraphy stage after NAC. The results showed an IR of 92.9% for SLNs with an FNR of 14.0%. SLNB demonstrated a sensitivity of 86.0%, an accuracy of 94.9% and a negative predictive value of 92.7%.

One of the largest patient populations was examined in the GANEA2 study conducted by Classe et al. [39]. The authors aimed to evaluate the accuracy and safety of SLNB after NAC in 957 patients diagnosed with early BC. Before NAC, patients with cytologically proven node involvement were allocated to the pN1 group, consisting of 589 individuals, while the other patients were allocated into the cN0 group (307 individuals). The authors separately calculated the IR for each group, obtaining an IR of 97.6% for the cN0 group and an IR of 79.5% in the pN1 group. The overall FNR obtained by the authorial group was 11.9%.

In their study, Berberoglu et al. [40] examined the value of gamma probe-assisted intraoperative SLN evaluation in patients who received NAC for BC and underwent surgical treatment. They obtained an SLN IR of 92.6% in the 87 analysed tumours, with an FNR of 10.3%. The sensitivity, specificity, positive predictive value, negative predictive value and accuracy rates were 80.4%, 100%, 100%, 82% and 90%, respectively. The authors concluded that the method yielded a high diagnostic performance in terms of predicting axillary breast cancer metastasis, particularly macro-metastasis.

Another study that aimed to explore the role of sentinel lymph node biopsy (SLNB) in breast cancer patients who become cN0 after neoadjuvant chemotherapy was conducted by Damin et al. [41]. They investigated if ALND can be safely omitted after a negative SLNB in patients who shift from clinically positive to negative nodes after NAC. Their SLNB IR was 93.2%. The results also showed that after a follow-up of 55.8 months, 2.6% of patients subjected to SLNB without additional ALND experienced axillary recurrence, compared to 3.2% in the ALND group. Also, in this group, the distant recurrence was more prevalent. Patients not undergoing ALND demonstrated notably enhanced overall survival and disease-free survival.

In their prospective study, Bordea et al. [26] aimed to evaluate the accuracy of SLNB after NAC and identify clinical and pathological factors that correlated with SLN invasion. The analysis included 47 consecutive breast cancer patients at stages II B-III A who underwent NAC and achieved a complete axillary response. Their results were highly promising, with a sensitivity of 91%, a specificity of 100%, a positive predictive value of 100%, a negative predictive value of 93% and an overall method accuracy of 96%. Furthermore, the FNR obtained in their population was just 4%.

The primary aim of the research conducted by Dalus et al. [24] was to assess the diagnostic value of lymphatic mapping through lymphoscintigraphy in BC patients who underwent NAC. The nuclear medicine procedure successfully identified at least one SLN in 55 patients, achieving an impressive IR of 90%. The SLNB IR in patients who underwent NAC was 86%, with FNR being 9.3%. The obtained overall accuracy of SLNB was 94%. In the subset of clinically node-negative patients, pathological examination revealed SLN metastases in 29% of cases.

In their study, Aragon-Sanchez et al. [42] retrospectively analysed the accuracy of SLNB after neoadjuvant chemotherapy in 85 patients who were initially cN1 and converted to cN0 following treatment. Their research revealed that SLNB demonstrated an adequate IR of 92.9%, but with a relatively high FNR of 19.1%. The authors stated that when excising more than three lymph nodes, the FNR diminished to 8.7%, improving the diagnostic accuracy.

The inclusion criteria were met by 20 papers, which were finally included in this qualitative review. Most researcher groups (n = 11) used a combination of ^99m^Tc-labelled radioisotopes and BD for their SLN mapping [24,28,29,30,31,32,37,38,39,40,41], while nine studies performed SLN identification through radioisotopic mapping alone [9,18,26,33,34,35,36,42]. In all papers, SLNB was followed by ALND to ensure the accurate assessment of the axilla.

The lowest IR for SLNB of just 79.5% was discovered by Classe et al. [39], with the highest IR (98%) appraised by Newman et al. [31]. Regarding the FNR, the lowest value was discovered by Tafra et al. [28], i.e., 0%, with Park et al. [35] revealing the highest FNR, i.e., 22%. The rates obtained by the other authorial groups ranged between these two values.

## 4. Discussion

NAC has become a standard treatment for locally advanced BC patients in the past few years. The protocol has been increasingly utilised for those with operable earlier-stage disease [43], with one of the most important benefits of it being its ability to downstage the disease, allowing a less extensive surgical procedure [44,45,46,47]. However, this practice has raised challenges in clinical decision-making, particularly concerning the identification and biopsy of the sentinel node, as false-negative results have been observed [18].

SLNB has been named as “the gold standard” for axillary staging in early BC patients with clinically negative lymph nodes, resulting in significant morbidity decrease, as well as life quality improvement without the loss of diagnostic accuracy and prognostic information [48,49]. With the increasing use of NAC, questions have arisen about the accuracy of SLNB in this particular context, especially in patients with larger tumours, but several trials have demonstrated that SLNB can be safely performed after NAC in these patients [15,50,51].

There are several performance parameters that need to be considered when analyzing the potential use of SLNB in patients with advanced-stage BC, with the most important being the identification rate and false-negative rate. In our patient population, we obtained an identification rate of 94.11%, which is in agreement with the IR obtained by the other authors. In the studies included in our review, the IR ranged between 79.5 and 98% [31,39].

Technical factors have been shown to impact SLN identification rates in multiple studies. Dual-agent mapping has been demonstrated to significantly improve SLN identification rates. In the ACOSOG Z1071 the IR using dual agents was 93.8%, and it was only 88.9% when a single radiolabelled agent was involved, with the use of BD alone being the technique with the lowest SLN IR (78.6%) [23]. The same statement was made by Kuehn et al. [19] in their SENTINA study, who discovered that the additional use of BD was associated with a significant increase in the detection rate (87.8% vs. 77.4% with radioisotope alone) in arm C, resulting in a higher number of nodes being detected. In a meta-analysis performed by Mok et al. [52] in 2019, the pooled IR for dual-tracer SLN mapping (RI + BD) was 96.7%, a value extremely close to their obtained pooled IR for radioisotope mapping alone, i.e., 96.5%. Furthermore, they reiterated that the lowest detection rate for a SLN mapping method was for the use of BD alone, with the pooled IR for this technique being 86.8% [50]. In the research groups included in our review that only performed SLN mapping through radioisotopic method, the IR varied between 80.8%, obtained by Pecha et al. [33], and 97.8%, attained by Bordea et al. [26], which is once again in concordance with our results.

Regarding the FNR, the same meta-analysis by Mok et al. [52] states a pooled FNR value for RI method alone of 2.6%, which, surprisingly, increases to 5.5% when dual-tracer method is used, but this result is probably due to the smaller number of studies that analysed the RI technique included in their pooled analysis compared to the ones performed using RI + BD. The studies including the largest patient populations obtained higher values for the RI method compared to RI + BD. The SENTINA study described an overall FNR value for arm C of 14.2%, with an FNR for ^99m^Tc-based mapping of 16%, which decreased to 8.6% when dual mapping was involved [19]. ACOSOG Z1071 reiterates these findings, describing FNRs for BD, RI and RI + BD of 21.4%, 8.6% and 6.2%, respectively [23]. When performing SLNB + ALND, the FNR that we obtained in our patient cohort was 7.9%, which is close to that observed in the ACOSOG Z1071 study and in agreement with those obtained by the other authorial groups and the generally accepted FNR of 10% [19]. The range of FNR in the reviewed studies varied between 0%, observed by Tafra et al. [28], and 22%, in the study performed by Park et al. [35].

Radioisotope-based SLN mapping offers good detection prospects with low FNR, whether as a solitary method or in combination with BD, but other mapping techniques, such as indocyanine green, superparamagnetic iron oxide and contrast-enhanced ultrasound imaging, have lately gained widespread recognition. Of these three methods, indocyanine green is considered to be as the most reliable, with a pooled IR of 97.9% and an FNR of 0.6%, according to the meta-analyses performed by Mok et al. [52]. In a recent phase II trial, Jung et al. [53] described using a dual method, combining indocyanine green and ^99m^Tc-lebelled radiotracers, for SLN mapping in BC patients who underwent NAC prior to SLNB and compared this method to RI alone. They observed that the IR in the group mapped with the aforementioned dual method was 98.3%, being higher than the one obtained in the RI-alone group, where IR was 93.8% [53]. However, when comparing the two SLN mapping agents alone, the IRs for both of them were relatively similar, with an RI for indocyanine green of 94.7% and for 99mTc-lebelled radiotracers of 93%; their results highlighted the superiority of dual-tracer methods without diminishing the value of RI technique [53].

The safety and accuracy of SLNB in clinically node-positive patients are still being evaluated through ongoing research. Traditionally, axillary lymph node dissection has been the standard treatment for cN1/2 patients, but new studies show promising results in these patients, encouraging this approach. In these cases, SLNB alone emerges as an appealing alternative, as it can help to avoid the morbidity associated with ALND, such as seroma formation, arm lymphedema and shoulder dysfunction, which have been reported in up to 44% of cases [54,55,56].

Considering the treatment response in patients who received NAC, several studies indicated that positive lymph node axilla can be converted to pathologically negative status with the use of NAC in about 32–74% of patients [9,19,30,42,51,57]. Newman et al. [31] described a ycN0 conversion rate of 32%, while Dominici et al. [58] reported a pathologic complete response rate of 74%, observed in the 109 women included in their study. In our research, we discovered a pathologically complete response rate of 77.36%, which was higher than those presented in the other papers, but probably correlated to the various therapeutic schemes that our patient population underwent.

The results of trials such as ACOSOG Z0010 and Z0011 indicate that SLNB for axillary lymph node staging exhibits similar outcomes to ALND in terms of relapse rates [59,60,61]. In a tumour-free SLN after SLNB, the likelihood of cancer cells having spread to the remaining axillary nodes is considered to be less than 10% [61]. Furthermore, the long term-results of the ACOSOG Z0011 randomised trial describe a cumulative incidence of nodal recurrences at 10 years of 0.5% for ALND and 1.5% for SLNB alone, with a ten-year cumulative locoregional recurrence of 6.2% with ALND and 5.3% with SLNB alone [61]. These findings suggest that performing SLNB alone is safe, allowing the omission of completion ALND, which can be beneficial for patients. Furthermore, the studies performed by Galimberti et al. [18] and Tinterri et al. [57] regarding the follow-up of patients who became or remained cN0 after NAC and underwent SLNB-alone confirm that the status of SLN remains a significant prognostic factor in these patients. In our patients, after a median follow-up of 31.3 months, recurrence was observed in four patients, resulting in a distant disease-free survival rate of 98%.

There are, however, several limitations to our study. Firstly, due to the retrospective nature of our research, data collection was challenging, resulting in a relatively limited number of enrolled patients. Secondly, another limitation of our study might be that not all patients received ALND, which was reserved for patients with suspected lymphatic tumoural invasion during intraoperative assessment of the axilla or who presented no SLN identification via lymphoscintigraphy or during surgery, which may represent an impediment when calculating the FNR. Nevertheless, FNR was only appraised in the group of patients who underwent both SLNB and ALND, and its result was in agreement with those obtained by the other authors.

## 5. Conclusions

In conclusion, the results of our study encourage the use of SLNB in breast cancer patients who undergo neoadjuvant chemotherapy, followed by radioisotopic mapping, for sentinel lymph node identification. SLNB accurately determines the lymph node status, with low FNR, and maintains its expected prognostic role with low recurrence rates in cN0-ycN0 patients. Our findings were, furthermore, supported by the results obtained in the studies included in our review. Overall, SLNB proves to be a valuable and reliable procedure in breast cancer patients treated with neoadjuvant chemotherapy, with a low recurrence rate in our study population.

## Figures and Tables

**Table 1:** Patients characteristics before NAC.

	cN0	cN1/2
Number of patients	47	55
Age (years)		
<35	2	2
35–49	17	15
50–64	13	20
>65	15	18
Clinical T		
cT1	9	0
cT2	35	30
cT3	3	24
cT4	0	1

Abbreviations: cN—clinical lymph node status; cT—clinical tumoural status.

**Table 2:** Stage of disease distribution before NAC.

	Number of Patients
Stage I	9
Stage II	65
Stage III	27
Stage IV	1

**Table 3:** Patients’ immunohistochemical characteristics.

	cN0	cN1
Grading		
G1	10	9
G2	26	33
G3	11	13
Immunohistochemical markers		
ER+	41	42
ER−	6	13
PR+	37	41
PR−	10	14
HER2+	11	20
HER2−	36	35
Triple negative	3	2

Abbreviations: ER—estrogen receptor; PR—progesterone receptor; HER2—human epidermal growth factor 2 receptor.

**Table 4:** Changes in tumoural staging after NAC in relation to the initial stage.

	after NAC				
Before NAC	Stage	0	I	II	III
	I	2	5	2	3
	II	13	30	22	1
	III	5	13	4	4
	IV	0	1	0	0

Abbreviations: NAC—neoadjuvant chemotherapy.

**Table 5:** Pathological response to NAC based on initial axillary stage.

	N0	N1/2	Total
Complete response	8 (17%)	12 (21.8%)	20 (19.6%)
Partial response	20 (42.5%)	29 (55.72%)	49 (48.03%)
Stable disease	17 (36.17%)	13 (23.63%)	30 (29.41%)
Progression	2 (4.25%)	1 (1.81%)	3 (2.94)
Total	47	55	102

Abbreviations: N0—no lymph node involvement; N1/2—lymph node involvement.

**Table 6:** Histopathological results after SLNB based on initial axillary stage.

	N0	N1
Micrometastasis (<2 mm)	6	5
Macrometastasis (>2 mm)	1	4
Micro- and macro-metastasis	0	3
No metastases	40	43

**Table 7:** Characteristics of studies that have researched the role of lymphoscintigraphy after NAC in BC patients.

No.	Study	Year	No. Patients	Stage of Disease	Lymph Node Involvement Prior to NAC	SLN Mapping Technique	RPh	Surgery Technique	IR (%)	FNR (%)
1	Tafra et al. [28]	2001	29	NA	cN0–N1/2	RI + BD	^99m^Tc sulphur colloid	SLNB + ALND	93	0
2	Shimazu et al. [29]	2004	47	T2–T4	cN0–N1/2	RI + BD	^99m^Tc-tin colloid	SLNB + ALND	94	12.1
3	Kinoshita et al. [30]	2007	104	T2–T4	cN0–N1/2	RI + BD	^99m^Tc-phytate colloid	SLNB + ALND	93.4	10
4	Newman et al. [31]	2007	54	NA	NA	RI + BD	^99m^Tc sulphur colloid	SLNB + ALND	98	8.6
5	Ozmen et al. [32]	2009	77	T1–T4	cN0–N1/2	RI + BD	^99m^Tc sulphur colloid	SLNB + ALND	92	13.7
6	Pecha et al. [33]	2011	343	T1–T4	cN0–N1/2	RI	^99m^Tc-labelled nanocolloid albumin	SLNB + ALND	80.8	19.5
7	Canavese et al. [34]	2011	64	T1–T4	cN0–N1/2/3	RI	^99m^Tc-labelled nanocolloid albumin	SLNB + ALND	93.8	5.1
8	Park et al. [35]	2013	178	T1–T3	cN0–N1/2	RI	^99m^Tc-phytate colloid	SLNB + ALND	94.9	22
9	Lee et al. [36]	2013	96	T1–T3	cN0–N1/2/3	RI	^99m^Tc tin colloid	SLNB + ALND	84.3	18.4
10	Kuehn et al. [19]	2013	592 (arm C)	NA	cN+	RI + BD	NA	SLNB + ALND	80.1	14.2
11	Yagata et al. [37]	2013	95	T1–T4	cN0–N1/2	RI + BD	^99m^Tc-phytate colloid	SLNB + ALND	85.3	15.7
12	Kim et al. [38]	2015	199	T0/is–T3	NA	RI + BD	NA	SLNB + ALND	95.8	10
13	Boughey et al. [23]	2015	756	T0–T4	N1/2	BD + RI	NA	SLNB + ALND	92.7	6.2
14	Andreis et al. [9]	2016	170	T2–T4	cN0–N1	RI	^99m^Tc-labelled nanocolloid albumin	SLNB + ALND	92.9	14
15	Classe et al. [39]	2018	957	T1–T4	cN0–N1	RI + BD	NA	SLNB + ALND	cN0: 97.6 pN1: 79.5	11.9
16	Berberoglu et al. [40]	2019	91	T0–T4	cN1/2	RI + BD	^99m^Tc-labelled nanocolloid albumin	SLNB + ALND	92.6	10.3
17	Damin et al. [41]	2020	59	T1–T3	cN1/2/3	RI + BD	^99m^Tc-labelled colloid	SLNB + ALND	93.2	NA
18	Bordea et al. [26]	2021	47	T1–T3	cN0–N1	RI	^99m^Tc-labelled nanocolloid albumin	SLNB + ALND	97.8	4
19	Dalus et al. [24]	2021	61	T1–T4	cN0–N1/2/3	RI + BD	^99m^Tc-labelled nanocolloid albumin	SLNB + ALND	86	9.3
20	Aragon-Sanchez et al. [42]	2022	85	T1–T3	cN1	RI	^99m^Tc-labelled nanocolloid albumin	SLNB + ALND	92.9	19.1

Abbreviations: RPh—radiopharmaceutical; IR—identification rate; FNR—false-negative rate; RI—radioisotope; BD—blue dye; SLNB—sentinel lymph node biopsy; ALND—axillary lymph node dissection.

## Data Availability

All data generated or analysed during this study are included in the manuscript.
